# Quantitative and qualitative changes in platelet traits of sunitinib-treated patients with renal cell carcinoma in relation to circulating sunitinib levels: a proof-of-concept study

**DOI:** 10.1186/s12885-022-09676-0

**Published:** 2022-06-13

**Authors:** Bibian M. E. Tullemans, Sanne L. N. Brouns, Frauke Swieringa, Siamack Sabrkhany, Franchette W. P. J. van den Berkmortel, Natascha A. J. B. Peters, Peter de Bruijn, Stijn L. W. Koolen, Johan W. M. Heemskerk, Maureen J. B. Aarts, Marijke J. E. Kuijpers

**Affiliations:** 1 Cardiovascular Research Institute Maastricht, Department of Biochemistry, Maastricht University, Maastricht, The Netherlands; 2 Synapse Research Institute, Maastricht, The Netherlands; 3 Cardiovascular Research Institute Maastricht, Department of Physiology, Maastricht University, Maastricht, The Netherlands; 4 Department of Medical Oncology, Zuyderland Medical Centre, Sittard-Geleen, The Netherlands; 5 Department of Internal Medicine, Sint Jans Gasthuis, Weert, The Netherlands; 6 Department of Medical Oncology, Erasmus MC Cancer Institute, Erasmus University Medical Centre, Rotterdam, The Netherlands; 7 Department of Pharmacy, Erasmus University Medical Centre, Rotterdam, The Netherlands; 8 Department of Medical Oncology, Maastricht University Medical Centre+, Maastricht, The Netherlands; 9 Thrombosis Expertise Centre, Heart and Vascular Centre, Maastricht University Medical Centre, Maastricht, The Netherlands

**Keywords:** Platelet count, Platelet aggregation, Sunitinib, Tyrosine kinase inhibitor, Renal cell carcinoma, cancer, Patients, Plasma, Serum, Bleeding

## Abstract

**Background:**

Tyrosine kinase inhibitors (TKIs), such as sunitinib, are used for cancer treatment, but may also affect platelet count and function with possible hemostatic consequences. Here, we investigated whether patient treatment with the TKI sunitinib affected quantitative and qualitative platelet traits as a function of the sunitinib level and the occurrence of bleeding.

**Methods:**

Blood was collected from 20 metastatic renal cell carcinoma (mRCC) patients before treatment, and at 2 weeks, 4 weeks and 3 months after sunitinib administration. We measured blood cell counts, platelet aggregation, and concentrations of sunitinib as well as its N-desethyl metabolite in plasma, serum and isolated platelets. Progression of disease (PD) and bleeding were monitored after 3 months.

**Results:**

In sunitinib-treated mRCC patients, concentrations of (N-desethyl-)sunitinib in plasma and serum were highly correlated. In the patients’ platelets the active metabolite levels were relatively increased as compared to sunitinib. On average, a sustained reduction in platelet count was observed on-treatment, which was significantly related to the inhibitor levels in plasma/serum. Principal component and correlational analysis showed that the (N-desethyl-)sunitinib levels in plasma/serum were linked to a reduction in both platelet count and collagen-induced platelet aggregation. The reduced aggregation associated in part with reported bleeding, but did not correlate to PD.

**Conclusion:**

The sunitinib-induced reduction in quantitative and qualitative platelet traits may reflect the effective sunitinib levels in the patient. These novel results may serve as a proof-of-principle for other TKI-related drugs, where both platelet count and functions are affected, which could be used for therapeutic drug monitoring.

**Supplementary Information:**

The online version contains supplementary material available at 10.1186/s12885-022-09676-0.

## Background

In the past two decades, over 40 tyrosine kinase inhibitors (TKIs) have been developed and approved for the treatment of many cancer types [1]. The majority of these drugs target the receptor-linked tyrosine kinases for growth factors, e.g., receptors for vascular endothelial growth factor, and/or differentiation/proliferation factors (e.g., Flt, Fms, Kit, and Ret). Several other TKIs target intracellular tyrosine kinases (Abl, B-Raf, Btk, Itk, Src-family kinases and Syk). However, in spite of the intended specific targets, most TKIs used for treatment also have broad off-target effects, invariably affecting a range of protein tyrosine kinases. It is debated whether these off-target effects help to improve progression-free survival (PFS) and overall survival (OS) of the treated patients [2].

Until shortly, the survival of metastatic clear cell renal cell carcinoma (ccRCC) patients was poor with a 5-year survival rate of 12% [3]. However, in recent years treatment options have increased by the availability of immune- and targeted therapies, using anti-PD1 and anti-CTLA4 antibodies, as well as new TKIs [4–7]. Randomized controlled trials testing the targeted therapies showed an overall improvement in response rates, PFS and OS, especially for the intermediate and poor risk groups [4–7]. For the favorable risk patients, it appeared that sunitinib, a broad-spectrum TKI, was superior to the immuno-agents nivolumab/ipilimumab, and was equivalent to pembrolizumab plus axitinib [4, 5]. Therefore, sunitinib has become a mainstay not only for first-line treatment in this particular risk group, but also in second line treatment after nivolumab/ipilimumab.

Upon activation of platelets via non-G-protein-coupled receptors, tyrosine phosphorylation, via Src-family kinases and Syk, is one of the key signal transduction mechanisms [8]. Especially, platelet activation via the collagen receptor glycoprotein (GP) VI fully relies on this tyrosine kinase pathway. Clinical studies have shown that several TKIs, next to affecting platelet function, also affect platelet count. Both effects on platelets can contribute to the increased bleeding risk, regularly observed in patients treated with TKIs [1, 9]. Also for sunitinib treatment, mild bleeding diathesis has been described as a side effect. This was observed as epistaxis, or mucocutaneous and gastrointestinal bleeding, which occurred in approximately 19% of the sunitinib-treated patients [10].

Previously, we have demonstrated a rapid uptake of sunitinib by platelets, which was accompanied by a reduced collagen receptor-induced aggregation, secretion of α-granules and thrombus formation under flow conditions, both in vitro and ex vivo in RCC patients on treatment [11]. Furthermore, we and others have shown that the platelet concentration decreases upon sunitinib treatment [11–13], an effect that has been postulated as a prognostic factor for the sunitinib treatment response in RCC [12]. However, to which extent these sunitinib effects are related to each other and to the circulating sunitinib concentration and/or bleeding has not been studied thus far.

In the present study with 20 patients, we now investigated as a proof-of-concept with regards to other TKIs, whether the effects of sunitinib on quantitative (count) and qualitative (aggregation) platelet traits are associated with the circulating inhibitor level. Furthermore, we set out to deduce how this relates to reported bleeding symptoms.

## Materials and methods

### Materials

Sunitinib malate (Sutent) was provided by Pfizer (New York NY, USA). Bovine serum albumin (BSA), D(+)-glucose, and apyrase were purchased from Sigma-Aldrich (Saint Louis MO, USA). Horm collagen type I was from Takeda (Hoofddorp, the Netherlands). Ilomedin (iloprost) injection fluid was obtained from Bayer (Mijdrecht, the Netherlands).

### Study population and blood collection

The study was performed in accordance with the declaration of Helsinki and approved by the local medical ethical committee of Maastricht University Medical Center+ (MUMC^+^). Full informed written consent was obtained from all participants. Blood samples were collected from 20 patients diagnosed with metastatic renal cell carcinoma (mRCC) at the Department of Medical Oncology of MUMC+ (Maastricht, the Netherlands), Zuyderland Medical Centre (Sittard-Geleen, the Netherlands) and SJG Hospital (Weert, the Netherlands). Blood was also collected from 10 healthy donors of similar age and gender. Patients were included, when eligible for treatment with sunitinib as a single agent (50 mg/day) with a treatment schedule of 4 weeks on and 2 weeks off medication. Patient blood samples were collected at four different timepoints: (i) before start with sunitinib, and after (ii) 2 weeks, (iii) 4 weeks and (iv) 3 months of sunitinib administration.

Blood samples were obtained from the antecubital vein. At each timepoint; 10 ml was collected using a vacuum container containing 3.2% trisodium citrate, and a second blood sample of 10 ml was collected in a Clot Activator Tube (CAT) Serum Separator (Greiner Bio-One, Alphen a/d Rijn, the Netherlands). Blood cell counts and hematological parameters were assessed in patient and healthy control samples, using a Sysmex XP300 (Kobe, Japan).

### Reporting of bleeding

Patients were asked to fill out a self-assessment bleeding form after 2 weeks, 4 weeks and 3 months of sunitinib administration, as was used earlier [14]. The physicians provided additional information with regards to bleeding. Any bleeding was scored as 1, no bleeding was scored as 0.

### Response evaluation

A response to sunitinib treatment was defined as partial response (PR), stable disease (SD) or progressive disease (PD) according to Response Evaluation Criteria in Solid Tumors (RECIST) 1.1 guidelines, based on a CT scan performed 3 months after initiating sunitinib, as compared to the CT scan prior to treatment. For some of the 20 patients, values were only collected at timepoints 0 and 2 weeks. Missing values at 4 weeks and/or 3 months were due to dose reduction (in 3 patients, after severe side effects of medication), hospital admission as a result of thrombocytopenia requiring blood transfusion (2 patients) or death (4 patients).

### Platelet isolation

Platelets were isolated from whole blood, as described previously [15]. Platelet-rich plasma (PRP) was isolated from citrate-anticoagulated blood by centrifugation at 240 *g* for 15 minutes. The PRP was supplemented with 1:10 acidic citrate dextrose (ACD; 80 mM trisodium citrate, 52 mM citric acid and 180 mM glucose) and centrifuged for 2 minutes at 2230 *g*. The supernatant plasma was retained in case of patient samples, and was further processed, as described below. The platelet pellet was resuspended into Hepes buffer pH 6.6 (10 mM Hepes, 136 mM NaCl, 2.7 mM KCl and 2 mM MgCl_2_) supplemented with 5 mM glucose and 0.1% bovine serum albumin (BSA). After addition of 1:15 ACD and 1 U/mL apyrase, the platelets were centrifuged for 2 minutes at 2230 *g,* followed by resuspension into Hepes buffer pH 7.45 with 5 mM glucose and 0.1% BSA. Platelet count was determined using a Sysmex XP300 (Kobe, Japan) and adjusted as stated per assay.

### Patient blood sample preparations

Serum was isolated from CAT tubes by centrifugation at 2200 *g* for 10 minutes at room temperature. Both serum and plasma (obtained from PRP) were centrifuged a second time at 22,500 *g* for 5 minutes to remove possible debris. The collected plasma and serum samples were frozen, and stored at − 80 °C until further use.

Isolated platelets (250 × 10^9^ platelets/L) were incubated for 5 minutes at room temperature with 50 nM iloprost, and centrifuged for 2 minutes at 2230 *g*. Supernatant was discarded, and the pellet was frozen and stored at − 80 °C until further use.

### Light transmission aggregometry

Washed platelets (250 × 10^9^ platelets/L) were incubated for 5 minutes at 37 °C before stimulation. Aggregation response was induced by addition of 1 μg/mL collagen type I, except for patients with anti-platelet drugs where aggregation was induced by 5 μg/mL collagen. The collagen concentration was kept the same at all timepoints per patient. Platelet aggregation was recorded using a Chronolog optical aggregometer (Havertown PA, USA), and maximum aggregation amplitude was quantified at 8 minutes after collagen addition.

### Pharmacokinetic analysis of sunitinib in plasma and serum samples

Sunitinib and the N-desethyl metabolite (SU12662) levels were determined using quantified ultra-performance liquid chromatography/tandem triple-quadrupole mass spectrometry, as described previously [16] in plasma, serum and platelet pellets obtained from patients after 2 and 4 weeks on sunitinib treatment. The concentration of either compound in isolated platelets was corrected for the number of platelets in the pellet, and normalized to ng per 2.5 × 10^8^ platelets.

### Statistical analysis

Data are presented as median ± interquartile ranges. Datasets within patients were compared using the Wilcoxon matched pairs test, whereas comparisons to controls were determined using the Kruskal-Wallis test. Correlation analysis was performed using a nonparametric Spearman correlation (2-tailed) using GraphPad Prism 8 software. To identify associations within the dataset, variables were quantile normalized and a rotated principal component analysis, based on an eigenvalue over 1, was performed using the statistical package for social sciences (SPSS version 24). *P*-values less than 0.05 were considered to be statistically significant.

## Results

### Patient demographics and clinical characteristics

In the present study, 20 patients were included with mRCC, who were eligible for treatment with 50 mg sunitinib per day with a treatment schedule of 4 weeks on and 2 weeks off medication (Table 1). Median age of the mRCC patients was 65 years (range: 52-83). Of the patients, 6 were females (median age 65; range: 59-83) and 14 were male (median age 65.5; range: 52-80). All patients presented with ccRCC, except for two patients who were diagnosed with papillary RCC. Three patients required dose reduction of sunitinib during the study follow-up, due to side effects (Table 1). Four of the patients additionally received daily aspirin as an anti-platelet drug, one of whom received heparin during the 3-month follow-up. Two patients received the cholesterol-lowering drug atorvastatin, which can also affect platelet function [17, 18]. Furthermore, 10 healthy individuals were included with similar median age of 63.5 (range: 53-85), of whom 3 were female.

**Table 1:**
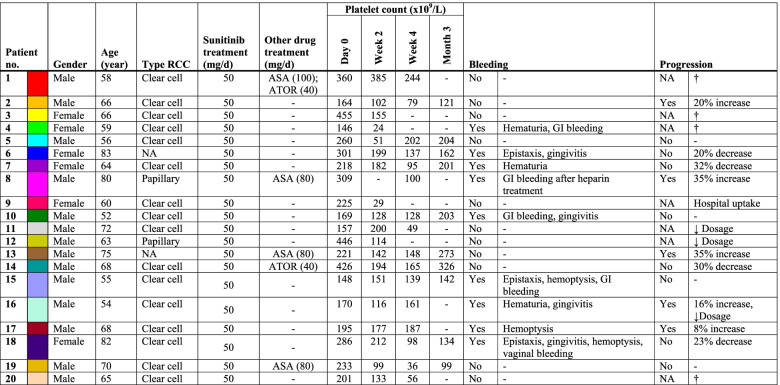
Demographics and clinical characteristics of RCC patients treated with sunitinib with a 3-month follow-up.

*ASA* Acetylsalicylic acid (aspirin), *ATOR* Atorvastatin, *GI* Gastrointestinal, *NA* Not available, *RCC* Renal cell carcinoma, *†* Patient deceased during follow-up period, ↓ *Dosage* Decreased dosage of sunitinib due to side effects.

### Plasma and serum levels of sunitinib and N-desethyl-sunitinib are highly correlated, while platelets take up more N-desethyl-sunitinib as compared to sunitinib

**Fig. 1:**
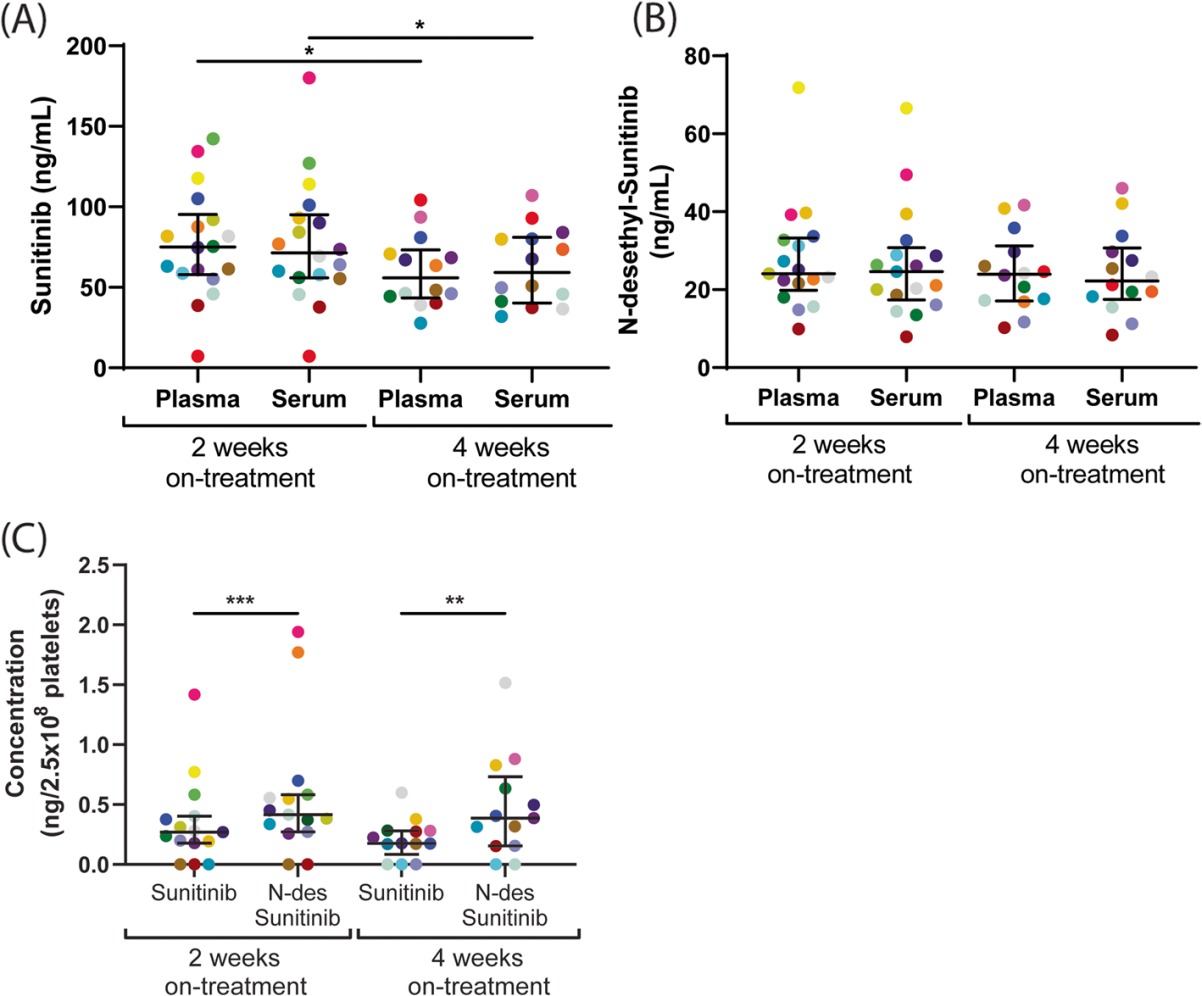
Comparison of sunitinib and N-desethyl-sunitinib levels in plasma, serum and isolated platelets from mRCC patients on 2 and 4 weeks on treatment. The concentration of sunitinib (**A**) and N-desethyl-sunitinib (**B**) were measured in plasma and serum samples obtained from mRCC patients at 2 and 4 weeks of sunitinib treatment. (**C**) The concentrations of both compounds were determined in isolated platelets, normalized to ng per 2.5 × 10^8^ platelets. Individual dots in the scatterplots represent one patient, for color coding see Table 1. Lines and error bars represent median ± interquartile range (*n =* 14-20). *P < 0.05. **P < 0.01, ***P < 0.001

Sunitinib and its active metabolite N-desethyl-sunitinib were determined in both plasma and serum from mRCC patients after 2 and 4 weeks of treatment using ultra-performance liquid chromatography/tandem triple-quadrupole mass spectrometry. The concentration of N-desethyl-sunitinib appeared to be significantly lower in serum and plasma than that of sunitinib (Fig. 1A vs 1B, plasma *P <* 0.0001 and serum *P <* 0.0006). Comparison of plasma and serum levels of sunitinib or N-desethyl-sunitinib did not show marked differences at 2 or 4 weeks (Fig. 1A, B). On the other hand, the concentration of sunitinib, but not of its metabolite, was slightly, but significantly, decreased after 4 weeks in both plasma and serum, as compared to 2 weeks on treatment (Fig. 1A). This is compatible with a 14-days peak level upon sunitinib administration, after which this reduced to a steady-state level.

Because platelets are known to sequester sunitinib, we also measured the concentrations of sunitinib and the active metabolite in isolated platelets from mRCC patients. Markedly, the concentration of the metabolite was significantly higher in platelets after 2 and 4 weeks on treatment, as compared to that of sunitinib (Fig. 1C). This contrasted to the lower concentration of the metabolite in the plasma and serum samples, suggesting a preferential uptake of the metabolite by platelets. Furthermore, the concentrations of sunitinib and metabolite after 2 and 4 weeks of treatment showed strong correlations between the plasma, serum and platelet samples (r > 0.67033, *P <* 0.01).

### Prolonged changes in platelet count by sunitinib treatment of mRCC patients are related to inhibitor levels in plasma and serum

**Fig. 2:**
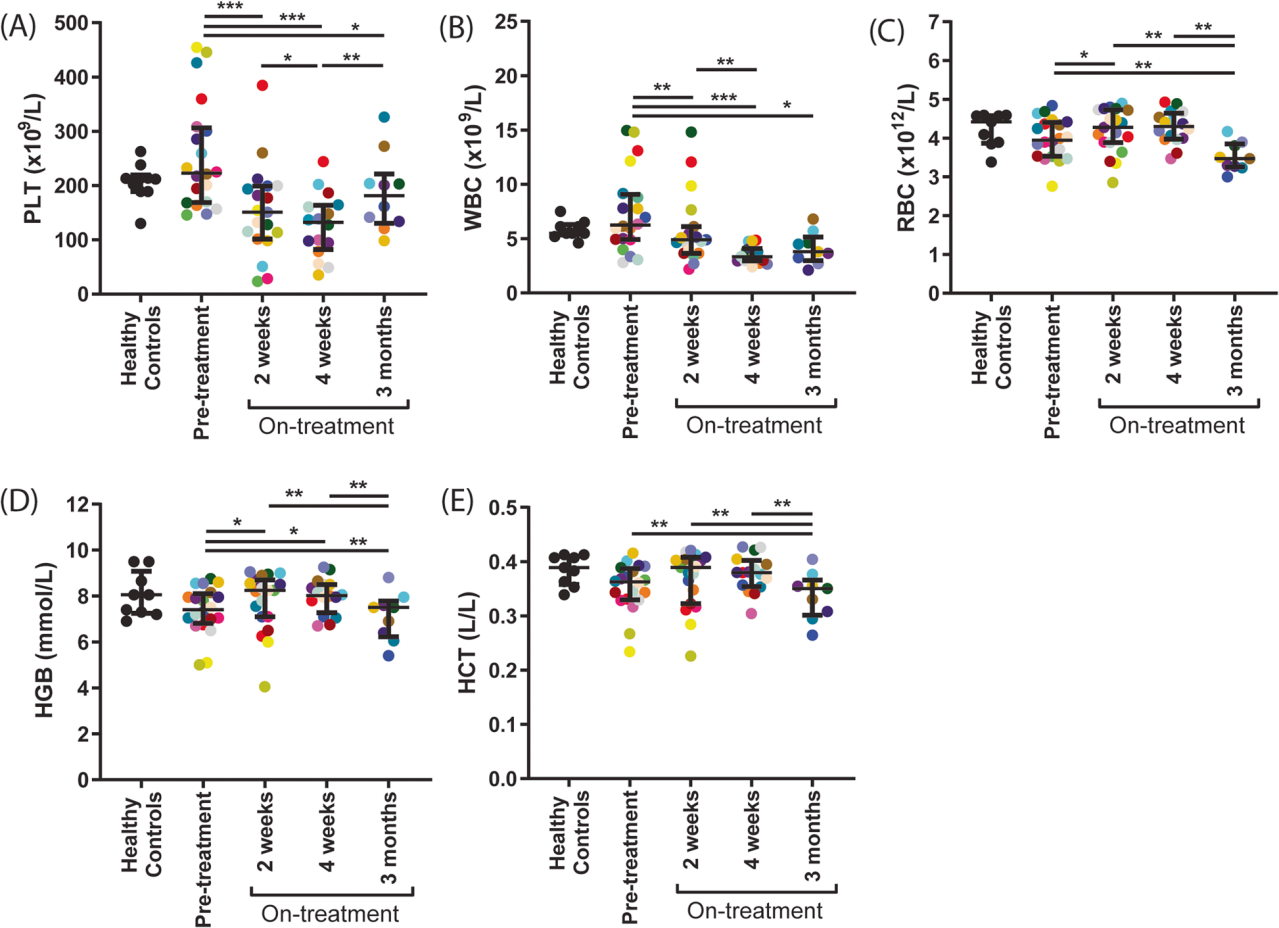
Treatment of mRCC patients with sunitinib decreases platelet count and other hematological parameters over time. Blood was collected from 10 healthy controls and 20 mRCC patients the day before, after 2 weeks, 4 weeks and 3 months on sunitinib treatment. Hematological parameters were measured in whole blood. Scatterplots show (**A**) platelet count (PLT), (**B**) white blood cell count (WBC), (**C**) red blood cell count (RBC), (**D**) hemoglobin concentration (HGB) and (**E**) hematocrit levels (HCT). Individual dots in the scatterplots represent one patient, for color coding see Table 1. Lines and error bars represent median ± interquartile range (*n =* 10-20). *P < 0.05. **P < 0.01, ***P < 0.001

Whole blood cell counts, hemoglobin and hematocrit were determined in blood samples from mRCC patients at four time points using a Sysmex cell counter (Fig. 2), i.e., the day before start of sunitinib treatment, after 2 and 4 weeks (during the first treatment cycle) and after 3 months (after 2 complete cycles). Before start of sunitinib treatment, no significant differences were observed in these parameters between patients and healthy controls (Fig. 2). However, after 2 weeks of treatment platelet counts were decreased (> 10%) in 15 patients (range: 17-87% reduction), while 5 patients showed no decrease or even an increase (5-10%, Fig. 2A). Seven of the patients who presented a reduced platelet count showed an even further reduction after 4 weeks, for six of them this effect persisted for 3 months. The median platelet concentration of all patients increased after 3 months as compared to 4 weeks, yet was still lower than before the start of treatment (Fig. 2A). In total, fifteen patients had a platelet count between 24 and 148 × 10^9^/L (Table 1), which was below the normal range (150-400 × 10^9^/L, according to the Dutch Society of Hematology, NVH). Of note, 5 patients presented with formal thrombocytopenia (< 50 × 10^9^ platelets/L) after 2-4 weeks of treatment (of which 2 required hospitalization and blood cell transfusion, resulting in a drop out from the study). White blood cell (WBC) counts decreased (> 10%) in 13 patients (range: 19-60% reduction) after 2 weeks of sunitinib treatment (Fig. 2B), which decrease persisted in most patients for 3 months. According to the Common Terminology Criteria 7 patients presented with grade 2 neutropenia during the study period with no more than 3 patients per time point. No patients had grade 3 or 4 neutropenia. After 2 weeks of sunitinib administration, overall red blood cell (RBC) count and hemoglobin levels were slightly, but significantly increased (Fig. 2C-D). After 4 weeks and 3 months, these values were decreased (compared to 2 weeks and to baseline), as well as the hematocrit (Fig. 2E).

**Fig. 3:**
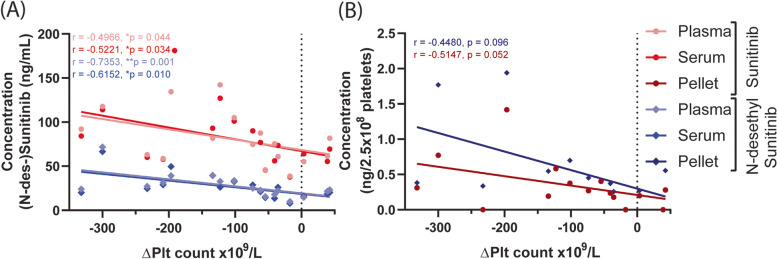
Concentrations of (N-desethyl-)sunitinib correlate with the reduction in platelet count. (**A**) Correlations between the concentration of sunitinib and N-desethyl-sunitinib in plasma and serum and difference in platelet count (ΔPlt count = Plt count at 2 weeks on treatment – platelet count before treatment). (**B**) Correlation between the concentration of sunitinib and N-desethyl-sunitinib in isolated platelets (normalized to ng/2.5 × 10^8^ platelets) and difference in platelet count

We examined whether the concentrations of (N-desethyl-)sunitinib were correlated with the reduction in platelet count. The levels of either compound in plasma and serum appeared to be correlated with the count reductions after 2 weeks treatment (Table 2, Fig. 3A), and for the metabolite this correlation was even stronger after 4 weeks (Table 2). For the compound levels in platelets, we noticed a borderline significant negative correlation (Table 2, Fig. 3B). Together, these data suggested that the changes in platelet count are related to the sunitinib levels in plasma and serum, thereby possibly reflecting the effective sunitinib dose in the patient.

**Table 2:** Concentrations of (N-desethyl-)sunitinib in plasma and serum correlate with changes in platelet count and aggregation in RCC patients at different time points

Variables		Time point	Change in platelet count				Correlation	
			-∆Plt median (IQR)	n	+∆Plt median (IQR)	n	R	***P***-value
Sunitinib	Plasma	Week 2	78.5 (60.5-108.3)	*n =* 14	61.4 (55.3-81.8)	*n =* 3	−0.4966	0.0443*
	Serum		80.6 (57.4-104.3)	*n =* 14	64.0 (55.4-69.4)	*n =* 3	−0.5221	0.0336*
	Pellet		0.29 (0.18-0.54)	*n =* 12	0.20 (0.00-0.28)	*n =* 3	−0.4480	0.0960
	Plasma	Week 4	56.0 (43.0-73.5)	*n =* 14	–	*n =* 0	−0.4066	0.1505
	Serum		59.3 (40.3-81.0)	*n =* 14	–	*n =* 0	−0.3846	0.1755
	Pellet		0.18 (0.09-0.28)	*n =* 13	–	*n =* 0	−0.2707	0.3701
N-desethyl-Sunitinib	Plasma	Week 2	26.0 (21.0-35.3)	*n =* 14	21.6 (14.8-23.2)	*n =* 3	−0.7353	0.0011**
	Serum		26.2 (18.6-34.3)	*n =* 14	18.6 (16.1-20.3)	*n =* 3	−0.6152	0.0100*
	Pellet		0.43 (0.35-0.67)	*n =* 12	0.27 (0.00-0.56)	*n =* 3	−0.5147	0.0517
	Plasma	Week 4	24.0 (17.0-31.5)	*n =* 14	–	*n =* 0	−0.7099	0.0058**
	Serum		22.3 (17.5-30.7)	*n =* 14	–	*n =* 0	−0.7143	0.0054**
	Pellet		0.39 (0.15-0.73)	*n =* 13	–	*n =* 0	−0.5557	0.0515
∆Aggregation		Week 2	−9.8 (−24.3 - -1.9)	*n =* 11	−5.7 (−6.6 - 35.6)	*n =* 3	0.6249	0.0192*
		Week 4	−7.1 (−44.7 - 4.4)	*n =* 13	–	*n =* 0	0.1099	0.7231
		Month 3	−6.6 (−17.3 - 0.3)	*n =* 10	−17.6 (− 18.1 - -17.1)	*n =* 2	−0.5515	0.1049

*IQR* Interquartile range, *ΔPlt count* Platelet count at indicated timepoint on treatment – platelet count before treatment, Negative value (−ΔPlt) indicates a reduction in platelet count; Positive value (+ΔPlt) indicates an increase in platelet count. Correlations are based on all changes in platelet count

### Comparative changes in quantitative and qualitative platelet traits by sunitinib treatment of mRCC patients

**Fig. 4:**
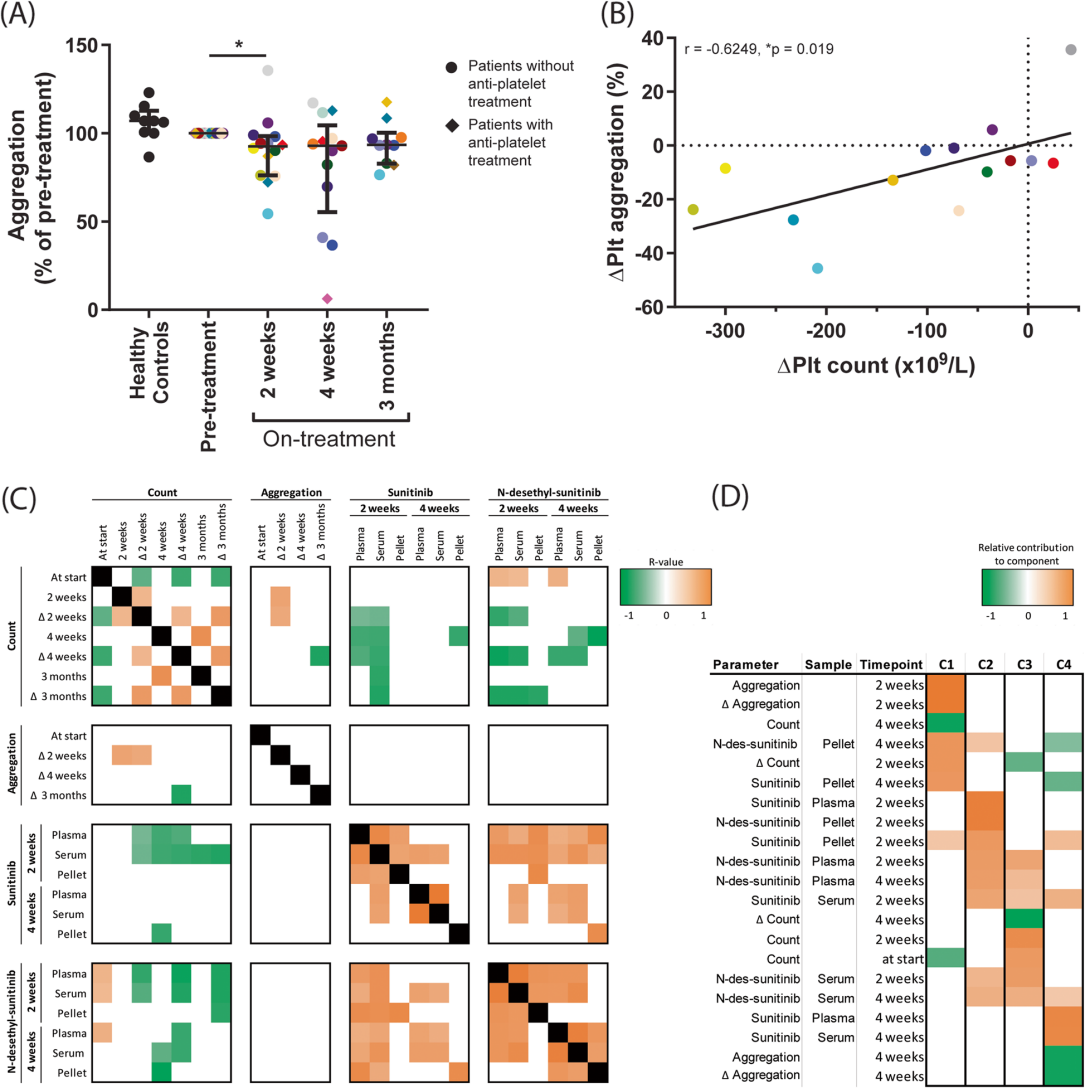
Platelet aggregation is reduced in RCC patients after 2 weeks of sunitinib treatment and correlates with reduction in platelet count. Blood was collected from 10 healthy controls and 20 RCC patients the day before, after 2 weeks, 4 weeks and 3 months on sunitinib treatment. **A** Light transmission aggregometry was induced in isolated platelets (250 × 10^9^/L) by collagen type I. Scatterplots indicate percentage of aggregation normalized to aggregation pre-treatment. Circles indicate platelets from patients treated with sunitinib, stimulated with 1 μg/mL collagen; diamonds indicate platelets from patients treated with sunitinib and anti-platelet drugs, stimulated with 5 μg/mL collagen. Individual dots in the scatterplots represent one patient, for color coding see Table 1. Lines and error bars represent median ± interquartile range (*n =* 10-20), *P < 0.05. **B** Correlation between difference in platelet count (ΔPlt count = Plt count at 2 weeks on treatment – platelet count before treatment) and difference in platelet aggregation (ΔPlt aggregation = Maximum amplitude at 2 weeks on treatment – maximum amplitude before treatment). Individual dots in the scatterplots represent one patient, for color coding see Table 1. **C** Correlation of significant R values for (changes in) platelet count, change in aggregation response and (N-desethyl-)sunitnib concentration parameters, with a highly negative correlation in green, a highly positive correlation in orange and no correlation in white. **D** Rotated component matrix determined by principal component analysis. Heatmap shows relative contribution of parameters to the four components (C1-4) with an eigenvalue over 1. Heatmap was filtered to only include values greater than 0.4 or less than − 0.4 as important contributors to the determined component. Colors refer to a highly negative contribution in green, a highly positive contribution in orange and no correlation in white

Aggregation of isolated platelets was determined by light transmission aggregometry. For adequate dosing, the collagen concentration required for maximal platelet aggregation was determined using washed patient platelets before the sunitinib treatment. Either 1 or 5 μg/mL collagen was used, dependent on intake of aspirin or atorvastatin (see Table 1). This dose was maintained for all follow-up measurements per patient. Platelet aggregation responses on treatment were compared to pre-treatment measurements for each patient. This resulted in an overall decrease in aggregation (range: 5-45% decrease) in 10 mRCC patients after 2 weeks sunitinib treatment (Fig. 4A), despite of the observed variation between patients. Platelet aggregation was continuously decreased in 10 patients (in 7 patients > 10%) after 4 weeks, while in 5 patients there was no decrease or even increased aggregation as compared to pre-treatment. Altogether, on average no differences in aggregation were observed after 4 weeks. After 3 months, again no differences in aggregation were observed, due to substantial variation. Remarkably, platelets from 6 patients showed a long-term reduction (> 10% decrease) in collagen-induced aggregation until 3 months, while platelets from other patients were not or only minimally affected during this time-period (Fig. 4A). The five mRCC patients who were treated with both sunitinib and aspirin and/or atorvastatin, revealed no significant differences in platelet aggregation, as compared to patients on sunitinib monotherapy.

We investigated whether an observed reduction in platelet aggregation was correlated to a reduction in platelet count at all time points (Table 2). It appeared that the difference in platelet count (ΔPlt = Plt count at 2 weeks on treatment - platelet count before treatment) correlated with the difference in aggregation (ΔPlt aggregation = maximum amplitude at 2 weeks on treatment - maximum amplitude before treatment) at 2 weeks on treatment only (Table 2, Fig. 4B). Next, we compared the reduction in platelet aggregation with the inhibitor levels in plasma, serum and platelets, though no correlation was observed between these parameters (Fig. 4C).

To further unravel the relationship between the quantitative and qualitative platelet traits and sunitinib levels in patients, we performed a principal component analysis (PCA). The resulting heatmap (Fig. 4D) indicates which parameters tended to cluster together per component. Four components were uncovered, which combined accounted for 95% of the variance of this data. It is observed that the parameters contributing to component 1 (C1) included aggregation, count and platelet concentration of (N-desethyl-)sunitinib at 2 and 4 weeks. C2 was composed of the parameters of plasma, serum and platelet levels of (N-desethyl-)sunitinib, and C3 showed the related count levels and metabolite concentrations in plasma and serum at 2 and 4 weeks. C4 revealed an association of aggregation and sunitinib levels in plasma and serum at 4 weeks. Altogether, this PCA underlined that the (N-desethyl-)sunitinib levels in either serum or plasma from mRCC patients associated with the reduction in both platelet count and aggregation, suggesting that the quantitative and qualitative platelet traits were jointly affected by the relative exposure to this TKI.

### Changes in quantitative and qualitative platelet traits by sunitinib treatment of mRCC patients in relation to reported bleeding and disease progression

Of the 20 mRCC patients treated with sunitinib, 9 reported bleeding complications during the 3-month follow-up. Events included minor nose bleeds (epistaxis) or bleeding gums (gingivitis), blood in urine (hematuria), stool (gastrointestinal bleeding) or mucus (hemoptysis) (Table 1). Interestingly, of the 5 patients who were on dual treatment of sunitinib and aspirin, only 1 patient (also treated with heparin) reported bleeding events during the first cycle of sunitinib treatment.

We analyzed whether the altered platelet count correlated with the reported bleeding. As shown in Suppl. Table 1, this was not the case. On the other hand, we observed that platelet function, as measured by collagen-induced aggregation, was negatively correlated with bleeding at 4 weeks on-treatment (*P* = 0.02, Suppl. Table 1), although the number of patients included in this analysis was relatively small (*n =* 4-8). At the other time points this correlation was not observed.

After 3 months on treatment, CT scans were evaluated for cancer response in 15 patients (Table 1). In 5 of these patients, progression of the disease was established, with an average of 23% increase in tumor size. In the other 10 patients no progression was observed (Table 1). In this limited group of patients, no correlation was observed between disease progression and reduction in blood cell counts or platelet aggregation (Suppl. Table 2).

## Discussion

In the present study with patients treated with sunitinib for mRCC, we observed for the first time that changes in platelet count were related to the inhibitor levels in plasma and serum. The reduction in platelet count may thus reflect the effective sunitinib levels in the patient, which may be used for therapeutic drug monitoring [19]. Furthermore, we observed a novel strong correlation between effects on platelet count and collagen-induced platelet aggregation, regardless of the treatment time point. This pointed to a common effect of this TKI on quantitative and qualitative platelet traits, which was confirmed by PCA. Next to RCC, sunitinib is approved for the treatment of gastrointestinal stromal tumor (GIST) and pancreatic neuroendocrine tumors. Furthermore, phase 3 clinical trials with sunitinib have been completed with positive outcome for non-small cell lung cancer [20, 21]. Hence, the results of the present study may also be applicable to other cancer types. In addition, platelet traits have been reported to be affected by other TKIs used for treatment of a large variety of malignancies [1, 9]. Also, other anti-cancer drugs can be taken up by platelets [22]. Therefore, the results of the present study with sunitinib may serve as a proof-of-concept with regards to platelet trait effects of other drugs.

Here, we made the novel observation that the patients on-treatment who showed a stronger inhibition in platelet aggregation (at normalized platelet count), also displayed a lower platelet count in whole blood. This can be explained by two separate mechanisms elicited by sunitinib, being *(i)* an effect on megakaryocyte level, culminating in a lower platelet count, and *(ii)* an effect on platelet function (aggregation), likely via an impaired tyrosine kinase-mediated signaling. Regarding the first mechanism, sunitinib may affect megakaryocytes or precursor cells directly in the bone marrow via interference in the proliferation of megakaryocytes and the production of proplatelets, which processes are dependent on tyrosine kinase activity [23]. Alternatively, the effects on megakaryocytes may be indirect via altered thrombopoietin production. Tumors can stimulate platelet production via de secretion of various cytokines, especially interleukin-6, which can in turn enhance thrombopoietin expression in the liver [24]. A direct effect of sunitinib on tumor proliferation may result in reduced levels of thrombopoietin and thereby lowering of the platelet count. Besides platelet count, we confirmed that sunitinib also reduced the WBC count (up to grade 2) in the mRCC patients. Furthermore, also the RBC count, and hemoglobin and hematocrit levels were affected upon sunitinib treatment. These results point to an effect of sunitinib on different hematopoietic cell populations in the bone marrow. Other studies with larger patient numbers indeed concluded that a reduced neutrophil count could be a predictor of PFS and OS probability [13, 25]. With regard to RBCs, sunitinib has been reported to affect erythropoiesis in both directions, resulting in either anemia [26] or in erythrocytosis [27]. Markedly, in our small patient group, we observed a transient increase in RBC count, hemoglobin and hematocrit during the first cycle of sunitinib treatment, as reported previously and explained by cycling kinetics during the dosing schedule [28].

Regarding the second mechanism of sunitinib affecting tyrosine kinase-mediated platelet signaling, we observed that collagen-induced platelet aggregation was reduced in mRCC patients after 14 days of sunitinib treatment. Others have shown that this inhibition was already present after 24 hours of sunitinib treatment [29]. Besides aggregation, sunitinib also inhibits collagen-induced thrombus formation, exposure of phosphatidylserine under flow, as well as α-granule secretion [11, 29]. Recently, we showed on a mechanistic level that sunitinib inhibited the phosphorylation of 34 tyrosine kinases upon GPVI stimulation, as well as intracellular calcium responses [30]. Various tyrosine kinases were directly linked to GPVI signaling, such as Syk and Src family kinases. Yet, most tyrosine kinases were probably activated by agonists released from the α-granules upon GPVI activation, such as fibrinogen, activating integrin αIIbβ3 resulting in FAK1/2 phosphorylation, as well as Gas6, stimulating Tyro3/Sky. As platelet granules contain many molecules, also other non-ITAM-linked tyrosine kinases were phosphorylated upon GPVI stimulation via secondary events. This was in line with previous studies that showed that the protein tyrosine kinases that were affected by sunitinib are c-Src [29], as well as other kinases expressed in platelets (e.g., Axl, CSF and Itk) [1].

As platelets are able to take up sunitinib [11], we hypothesized that this affects the outcome of sunitinib plasma measurements. Therefore, we compared the concentrations of sunitinib and N-desethyl-sunitinib (active metabolite of sunitinib) in plasma, serum and isolated platelets from mRCC patients at 2 and 4 weeks of treatment. The concentration of N-desethyl-sunitinib was much lower than sunitinib in both serum and plasma, which is in line with previous results [31]. Furthermore, there was no difference between serum and plasma levels of either sunitinib or N-desethyl-sunitinib at 2 or 4 weeks of sunitinib administration. Honeywell et al. also reported no differences for sunitinib in plasma as compared to serum for 5 patients on sunitinib treatment after 24 hours and 3 weeks [32]. In contrast, another study demonstrated lower concentrations of sunitinib in plasma as compared to serum in patients treated with sunitinib [29]. These studies demonstrated a high variation in sunitinib plasma concentrations, which could only partly be explained by patient- or medication-related factors [33]. This highlights the importance for further research of therapeutic drug monitoring for individual dosage adjustments, especially in cases of toxicity [19]. Interestingly, the concentrations of the active metabolite were significantly higher in isolated platelets from mRCC patients at 2 and 4 weeks as compared to that of sunitinib itself. The only difference between the two molecules is an ethyl (CH_2_-CH_3_) group that is removed from sunitinib by cytochrome P450 [34]. As this enzyme is not present in platelets, these results suggest preferential uptake of the metabolite by these cells. Overall, we concluded that although the concentration of the active metabolite in platelets was higher, the uptake of sunitinib in platelets did not affect the sunitinib concentration in plasma versus serum. The serum concentration of N-desethyl-sunitinib negatively correlated with platelet count at 4 weeks of treatment, suggesting that higher platelet counts result in a lower concentration of metabolite present in serum. Together with the observation that metabolite concentrations in isolated platelets were higher, this may suggest that platelets do not secrete sunitinib after uptake.

Mild bleeding was reported in 9 of the 20 investigated cancer patients on sunitinib. No correlation existed between the platelet count at any time point on treatment and the occurrence of bleeding during the 3-month study period. This agrees with earlier reports that showed that the platelet count is a poor predictor for bleeding [35, 36]. Hence, the reported bleeding events may be explained by an accompanying inhibition of platelet activation by sunitinib. We observed a correlation between platelet aggregation responses and bleeding during sunitinib treatment, meaning that stronger reduction in collagen-induced platelet aggregation was observed in patients that reported bleeding episodes. Of note, only a small number of patients was included in this analysis, and at no other time points a correlation was observed between platelet aggregation and reported bleeding. Also, Walraven et al. did not observe this correlation between bleeding and platelet aggregation after 3 weeks of treatment in 5 patients [29]. As normal platelet functions can be considered to be required for effective control of hemostasis, correlations between bleeding and TKI-induced effects on platelet function should be investigated in larger clinical studies. Of note, in patients treated with sunitinib, also endothelial dysfunction and increased capillary leakage can be induced by treatment with sunitinib [37], which may also play a role in the increased bleeding tendency. Paradoxically, interfering with endothelial cell integrity can also shift the hemostatic balance in favor of thrombosis, and treatment with sunitinib and other TKIs have also been associated with an increase in arterial thromboembolic events in RCC patients [37, 38].

A correlation between platelet count and disease progression in this study could not be observed due to a relatively small number of patients. However, it has already been demonstrated in large, retrospective patient studies that a decrease in platelet count following sunitinib treatment more likely resulted in a response to therapy and longer OS in mRCC [12] and GIST [13]. In the present study we clearly showed that a reduction in platelet count is accompanied by an inhibition of platelet aggregation, which correlated to the (N-desethyl-)sunitinib levels in either serum or plasma of mRCC patients (component 1 of the PCA). Altogether this suggested that both quantitative and qualitative platelet traits were linked to the exposure to this TKI.

Renal cancer is a disease of the elderly, with most patients being diagnosed between 65 and 74 [39, 40]. Older cancer patients generally have more comorbidities, especially cardio-vascular disease, and therefore often receive anti-coagulant or anti-platelet drugs. In the present study, five patients were treated with sunitinib in combination with aspirin and/or atorvastatin, both affecting platelet functions [17, 18, 41]. In our tests, this did not result in significant differences in platelet aggregation as compared to patients on sunitinib without comedication. One explanation may be an increased platelet reactivity observed in cancer patients [42], which compensates for the intake of the anti-platelet drugs. On the other hand, a recent study did show that, sunitinib plus aspirin in vitro did further reduce platelet aggregation, thrombus formation and PS exposure on collagen under flow as compared to sunitinib alone [30]. This is in agreement with another study that showed combined effects of ibrutinib and aspirin on collagen-induced aggregation [43]. Therefore, the effects of dual anti-platelet and TKIs treatments on the inhibition of platelet function should be further investigated.

Our study encounters some limitations. Due to the limited number of patients and some missing values, no (clear) correlations were observed between platelet count and function on the one hand and bleeding and disease progression on the other hand. Also, only one platelet function test could be performed due to the limited amount of blood obtained from the patients. Out of multiple function tests, we opted for light transmission aggregometry as this is still the gold standard for platelet function testing. Due to ethical restrictions, the relation between platelet dysfunction after sunitinib treatment and abnormal (pro) platelet formation from megakaryocytes in the bone marrow could not be assessed. For the TKI dasatinib it has been reported that it promoted megakaryocyte differentiation, while it impaired migration and proplatelet formation [44]. The effects of TKI treatment on megakaryocyte proliferation and function should therefore be investigated in future studies.

## Conclusion

In this study with mRCC patients treated with sunitinib a novel strong correlation was found between the effects on platelet count and inhibition of aggregation as well as (N-desethyl-)sunitinib levels in plasma and serum. The effects on count and function may thus reflect the relative exposure of the patient to sunitinib. This pointed to an association between the effect of this TKI on quantitative and qualitative platelet traits. These results may serve as a proof-of-principle with regards to other drugs, indicating that TKI treatment can affect both platelet count and function, which could be used as a measure for relative drug exposure and therapeutic drug monitoring.

## Supplementary Information

**Additional file 1.**

## Data Availability

The data that support the findings of this study are available on request from the corresponding author. The data are not publicly available due to privacy or ethical restrictions.

## Abbreviations

ASA: Acetylsalicylic acid (aspirin)
ATOR: Atorvastatin
ccRCC: Clear cell renal cell carcinoma
CRP: Collagen-related peptide (in cross-linked form)
GI: Gastrointestinal
GIST: Gastrointestinal stromal tumor
GPVI: Glycoprotein VI
mRCC: Metastatic renal cell carcinoma
OS: Overall survival
PCA: Principal component analysis
PD: Progression of disease
PFS: Progression-free survival
PRP: Platelet-rich plasma
RBC: Red blood cell
TKI: Tyrosine Kinase inhibitor
WBC: White blood cell
