# Supplementary material for: Quantitative and qualitative changes in platelet traits of sunitinib-treated patients with renal cell carcinoma in relation to circulating sunitinib levels: a proof-of-concept study
Source: BMC Cancer. 2022 Jun 13;22:653. doi: 10.1186/s12885-022-09676-0 (PMC9195440; doi:10.1186/s12885-022-09676-0)
Supplement: Supplementary file 1 — Additional file 1. [file 12885_2022_9676_MOESM1_ESM.pdf]

Supplementary Material

Quantitative and qualitative changes in platelet traits of sunitinib-treated patients with renal cell carcinoma in relation to circulating sunitinib levels: a proof-of-concept study

Bibian M.E. Tullemans et al.

Supplemental Tables

Supplemental Table 1 Reported bleeding correlates with platelet aggregation in RCC patients at 4 weeks on treatment

Variables	Time point	Reported bleeding				Correlation	
		No median (IQR)	n	Yes median (IQR)	n	R	P-value
Platelet count	Week 2	132.5 (75.8-229.8)	n=9	164.0 (119.0-194.8)	n=8	-0.0515	0.8498
	Week 4	78.5 (49.0-202.0)	n=7	132.3 (98.4-155.0)	n=8	0.0201	0.9508
	Month 3	162.5 (104.5-255.8)	n=4	161.5 (137.8-202.0)	n=5	-0.3651	0.3786
ΔAggregation	Week 2	-10.7 (-29.6 - 4.0)	n=6	-3.8 (-6.7 - 0.8)	n=6	0.2612	0.4072
	Week 4	-3.8 (-5.7 - 12.2)	n=4	-24.0 (-62.3 - -7.8)	n=8	-0.6680	0.0206*
	Month 3	-10.2 (-22.1 - 12.7)	n=4	-6.8 (-12.0 - -4.8)	n=5	0.02623	0.9516
Progression %	-	10.0 (0.0 - 31.3)	n=4	0 (-22.3 - 14)	n=8	-0.2500	0.4285

IQR: interquartile range

Supplemental Table 2 No correlation of disease progression with changes in blood cell counts and platelet aggregation in mRCC patients

Variables	Time point	Progression				Correlation	
		No (PR+SD) median (IQR)	n	Yes (PD) median (IQR)	n	R	P-value
ΔPlatelet count	Week 2	-87.5 (-189.9 - -36.8)	n=8	-36.5 (-60.3 - 24.8)	n=4	0.4609	0.1535
	Week 4	-143.3 (-194.6 - -45.1)	n=8	-73.0 (-147.0 - -8.8)	n=5	0.2535	0.4351
	Month 3	-77.8 (-137.8 - -8.6)	n=8	4.5 (-42.5 - 51.5)	n=2	0.4352	0.2667
ΔWhite blood cell count	Week 2	-1.68 (-4.04 - -0.08)	n=8	-0.68 (-2.05 - 1.26)	n=4	0.4104	0.1980
	Week 4	-3.65 (-5.03 - -2.02)	n=8	-2.65 (-3.18 - -0.83)	n=5	0.4655	0.1181
	Month 3	-3.70 (-5.70 - -1.35)	n=8	0.65	n=1	0.5477	0.2222
ΔRed blood cell count	Week 2	0.21 (0.2 - 0.33)	n=8	0.25 (-0.07 - 0.41)	n=4	0.1282	0.7152
	Week 4	-0.06 (-0.17 - 0.18)	n=8	0.08 (-0.01 - 0.35)	n=5	0.3803	0.2222
	Month 3	-0.93 (-1.73 - -0.50)	n=8	-0.87	n=1	0.1369	0.8889
ΔAggregation	Week 2	-7.7 (-24.0 - -1.2)	n=8	-5.7	n=1	0.0688	1.0000
	Week 4	-24.0 (-60.2 - -4.2)	n=8	-6.6 (-72.1 - 7.3)	n=4	0.2132	0.6095
	Month 3	-6.6 (-14.5 - 5.6)	n=6	-10.2 (-18.1 - -2.4)	n=2	-0.0870	0.8889

IQR: interquartile range; PR: partial response; PD: progressive disease; SD: stable disease.
